# Optical imaging and gene transfection potential of linear polyethylenimine-coated Ag_2_S near-infrared quantum dots

**DOI:** 10.55730/1300-0152.2709

**Published:** 2024-08-21

**Authors:** Altay SAVALAN

**Affiliations:** Department of Chemistry, Koç University, İstanbul, Turkiye. Department of Pharmaceutical Biotechnology, Hamidiye Faculty of Pharmacy, University of Health Science, İstanbul, Turkiye. Experimental Medicine Practice and Research Center (EMPRC), Validebağ Research Center, University of Health Science, İstanbul, Turkiye

**Keywords:** Ag_2_S near-infrared quantum dots (Ag_2_S NIR QDs), biocompatible, linear polyethylenimine (LPEI), green fluorescent protein (GFP) gene, theranostic nanoparticle, cancer therapy

## Abstract

**Background/aim:**

The application of biocompatible heavy metal-free and cationic Ag_2_S NIR quantum dots (QDs), which have intense luminosity in the 700–900 nm medical range, as a nonviral gene delivery system paves the way to overcome autofluorescence and easily track the delivery of genes in real time.

**Materials and methods:**

The newly developed small and colloidally stable 2-mercaptopropionic acid (MPA)-capped Ag_2_S aqueous quantum dots electrostatically complexed with linear polyethyleneimine (Ag_2_S@2MPA/LPEI) were investigated for the first time both as a strong fluorescent probe and as a vector for nonviral gene delivery for the highest tracking of the system and delivery of genes into the nuclei of different cancer cells. The synthesized Ag_2_S@2MPA/LPEI quantum dots demonstrated strong optical imaging properties and were used to deliver a green fluorescent protein (GFP) plasmid as a standard gene.

**Results:**

For Ag_2_S@2MPA/LPEI-pDNA nanoparticles, an N/P ratio of 20 was the ideal transfection efficiency. Ag_2_S@2MPA/LPEI was substantially more compatible with HEK 293T cells than the free 25-kDa linear polyethylenimine (LPEI). Next, the transfection efficiency evaluation of pGFP genes with synthesized Ag_2_S@2MPA/LPEI and LPEI in different cancer cells demonstrated their high potential as a theranostic cancer gene delivery system.

**Conclusion:**

This is the first instance of gene transfection and optical imaging carried out in vitro using Ag_2_S@2MPA/LPEI QDs. Overall, the newly synthesized highly biocompatible and trackable Ag_2_S@2MPA/LPEI QDs can be an effective and biocompatible theranostic system for cancer gene therapy.

## 1. Introduction

Biocompatible heavy metal-free and cationic Ag_2_S near-infrared quantum dots (NIR QDs) are becoming increasingly promising theranostic systems for biomedical applications, due to their unique physicochemical features, possessing excellent biocompatibility, ultralow solubility product constant, and the absence of harmful metals as Cd^2+^, Hg^2+^, Pb^2+^, and As^3−^ (which ensures the minimum quantity of Ag_2_S released into the biological environment) having low toxicity (Lu et al., 2018). QDs’ aqueous solubility is one of the most common problems that need to be overcome in order to make them applicable for therapeutic utilization in the treatment of cancer (Duman et al., 2015; Karakoti et al., 2015). Because cancer develops as a result of the accumulation of genetic abnormalities, it continues to be a major source of pain and death despite significant advancements in oncology. Gene therapy appears to be a novel and promising treatment option for cancer patients (Wong et al., 2017). However, these therapeutic genetic materials such as siRNA, miRNA, and plasmid DNA are unstable, have poor cellular uptake, and, due to their instability, they can be rapidly degraded by nucleases present in the bloodstream (Ma et al., 2011; Wong et al., 2017; Zhang et al., 2024). In order to deliver these genetic materials to the target sites and successfully translocate them through the cell membrane, there is a critical need for the development of suitable delivery systems (Sokolova and Epple, 2008). These gene delivery mechanisms ought to be able to direct these macromolecules to the site of action, shield them from nuclease breakdown, and increase their absorption by cells (Haider et al., 2005; Tian et al., 2013). Furthermore, these delivery systems should induce significanlty low immunogenicity or toxicity and should also have a low cost. Despite their superior ability to deliver and express genes in the targeted cells, the application of viral vectors such as retroviruses, lentiviruses, adenoviruses, and adeno-associated viruses (AAVs) as gene delivery systems is limited owing to their shortcomings, such as immunostimulation, small transgene size, high cytotoxicity, and high cost (Morille et al., 2008; Yin et al., 2014; Wong et al., 2017). As an alternative system, nonviral vectors based on lipidic or polymeric formulations have attracted more interest due to their multipurpose modifications, capacity to carry large inserts, low cost, and low immunogenic response (Mintzer and Simanek, 2009; Perevyazko et al., 2012; Yin et al., 2014). The most effective water-soluble synthetic vector for gene transfection is the polyethylenimine (PEI) macromolecule, which is one of the various polycationic agents used to create polyplexes. It can condense nucleic acids, shield them from degradation, and form precise nanosized complexes (Lungwitz et al., 2005; Huh et al., 2007; Morille et al., 2008). They can be produced in branched or linear shapes and at varying lengths (Lungwitz et al., 2005; Morille et al., 2008). Additionally, they release nucleic acid into the cytoplasm by using the proton sponge effect (Lungwitz et al., 2005; Mehta et al., 2024). According to Intra and Salem (2008), when injected intraperitoneally, linear PEI-pDNA constructs have shown a higher level of efficiency in vivo, although branching PEI-pDNA demonstrated more effectiveness in vitro (Intra and Salem, 2008). In another study, application of 25 kDa branched PEI or 22 kDa linear PEI increased the transfection efficiency of plasmids up to around 200- or 1000-fold higher with respect to liposomal formulations (Wiseman et al., 2003). The molecular weight (MW) of PEI is a crucial factor for efficient transfection and with a higher MW they are usually more effective for gene transfection. However, a higher molecular weight PEI will also have significant toxicity. Another important factor is the ratio of PEI nitrogen to pDNA phosphate (N/P), which has an impact on transfection efficiency (Duman et al., 2015; Massadeh et al., 2016). Therefore, the type, MW, and N/P ratio of the PEI are the critical factors that should be critically optimized during the preparation of an effective polyplex. Here, we investigated the efficacy of our recently developed small and colloidally stable 2-mercaptopropionic acid (MPA)-capped Ag_2_S QDs electrostatically complexed with linear polyethyleneimine (Ag_2_S@2MPA/LPEI) both as a strong fluorescent probe for the highest tracking and as a biocompatible and highly effective nonviral gene delivery vector for cancer cells.

## 2. Materials and methods

### 2.1. Materials

Silver nitrate (AgNO_3_), Dulbecco’s modified Eagle’s medium (DMEM), 4,6-diamidino-2-phenylindole dihydrochloride (DAPI), phosphate buffered saline (PBS) tablets, and 2-mercaptopropionic acid (2MPA) were obtained from Sigma-Aldrich. Alfa-Aesar provided the sodium sulfide (Na_2_S). Linear polyethylenimines (25 kDa) were purchased from Polysciences Inc. and dimethyl sulfoxide (DMSO) was purchased from VWR. Acetic acid, sodium hydroxide, and ethanol (EtOH) were obtained from Merck. Fetal bovine serum (FBS) was purchased from Capricorn, McCoy’s 5A (modified) medium was purchased from Gibco (Thermo Fisher Scientific, Inc., Waltham, MA, USA), and trypsin–EDTA were purchased from Hyclone (USA). LDS 798 near-IR laser dye (quantum yield reported as 14% in DMSO by the producer) was purchased from Exciton Inc. Paraformaldehyde (4%) was purchased from Chemcruz. Human cervical carcinoma (HeLa), mouse embryonic fibroblast MEF p53(−/−), human colon cancer (HCT 116 wt), and HCT 116 p53(−/−) cells were obtained from Gözüaçık Laboratory (Sabancı University, Turkey). pBI-MCS-EGFP plasmid was purchased from Addgene. The Endo-Free Plasmid Maxi Kit was purchased from Qiagen.

### 2.2. Preparation of 2MPA/LPEI-coated Ag_2_S NIR QDs

**Figure 1:**
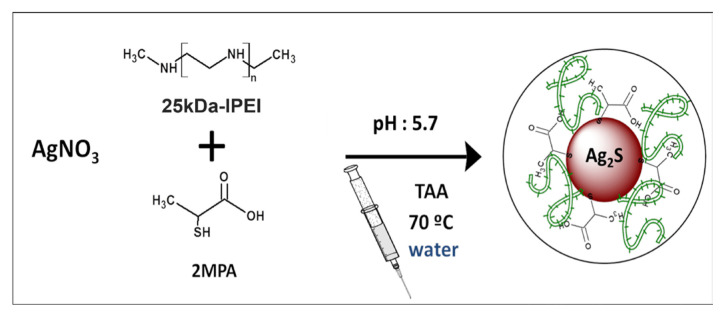
Schematic representation of the Ag_2_S@2MPA/LPEI synthesis.

Figure 1 depicts the schematic representation of the process used to create the 2MPA/L-PEI-coated Ag_2_S NIR QDs (Ag_2_S@2MPA/LPEI). First, a three-neck, round-bottomed flask containing 75 mL of deionized water was purged with argon for 15 min. The flask was filled with silver nitrate (0.25% mmol AgNO_3_), and the silver nitrate solution was then mixed with LPEI. The solution was then supplemented with the appropriate volume of 2-mercaptopropionic acid (2MPA). Acetic acid (CH_3_COOH) was used to bring the pH down to 5.7 following the addition of 2MPA. In a separate round-bottomed flask, 25 mL of deoxygenated water was used to dissolve thioacetic acid (TAA; 0.0625 mmol). The TAA solution was sonicated under argon for 15 min before being added to the reaction solution, using a syringe. Reactions were conducted at 70 °C for 3 to 5 h with a lot of mechanical stirring and argon. Aliquots were taken throughout the synthesis at various intervals to monitor the development of the particles. Ag_2_S QDs in a colloidal solution were stored at 4 °C in the dark after being cleaned with Milli-Q water in Amicon-Ultra centrifugal filters with a 3-kDa cutoff. The ratio of coating materials to Ag/S was fixed at 20/4/1. In these formulations, the moles of the thiol group in 2MPA and the secondary nitrogen in LPEI were employed. It was determined that the mole ratio of the 2MPA SH groups to the PEI N was 20/80.

### 2.3. Characterization

In the characterization studies, firstly, the organic percentage of synthesized QDs was determined using a thermogravimetric analyzer (TGA). Dynamic light scattering (DLS) analysis of the QDs was performed using a ZS Malvern Zetasizer. Then total nitrogen content was calculated using the organic CHNS/O elemental analysis method (Thermo Scientific Flash 2000).

### 2.4. Cell line and culture medium

Complete DMEM medium with 10% fetal bovine serum, 1% penicillin streptomycin antibiotic solution, and 4 mM L-glutamine was used to cultivate HeLa cells, human embryonic kidney (HEK 293T) cells, and MEF cells. Complete McCoy’s 5A medium, which contains 10% fetal bovine serum, 1% penicillin–streptomycin antibiotic solution, 1.5 mM L-glutamine, and 2200 mg/L sodium bicarbonate, was used to incubate HCT 116 wt and HCT 116 p53−/− cells. Under 5% CO_2_, all cell lines were cultured at 37 °C. Every 2 days, cell passage using fresh medium was carried out. Trypsin–EDTA was utilized for the cell detachment procedure.

### 2.5. Cytotoxicity analysis

Cell cytotoxicity analysis of Ag_2_S@2MPA/LPEI QDs and LPEI was conducted in HeLa, HCT 116 wt, and HCT 116 p53−/− cell lines by MTT assay. HeLa cells were grown 5 × 10^3^ cells/well in complete DMEM, whereas HCT 116 wt and HCT 116 p53−/− cells were grown 20 × 10^3^ cells/well in 96-well plates in McCoy’s 5A medium for 24 h. In summary, following a 24-h incubation period, Ag_2_S@2MPA/LPEI QDs and LPEI were introduced into each well using fresh medium at prescribed concentrations up to 200 μL. Following a 24-h treatment period, the medium in the wells was changed to 150 μL of the cell type-specific medium and 50 μL of MTT solution (5 mg/mL) was added. Only fresh medium was used to replace the medium in the control group. Following a 4-h incubation period with MTT reagent, the medium was disposed of and 200 μL of DMSO:EtOH (1:1) was applied to each well to dissolve the purple formazan result. The absorbance at 600 nm (ELx800 Biotek ELISA reader), which indicates the quantity of live cells, was used to calculate the formazan quantity.

### 2.6. In vitro cell internalization and optical imaging of Ag_2_S@2MPA/LPEI

Optical imaging of free Ag_2_S@2MPA/LPEI QDs was performed on HeLa cell lines. HeLa cells were cultured at a density of 5 × 10^4^ cells per well in 6-well plates using complete DMEM culture medium for 24 hours. The medium was then replaced with fresh DMEM containing 50 μg/mL Ag_2_S@2MPA/LPEI QDs. After 4 h of incubation, the medium was removed, and the cells were washed three times with PBS. The cells were then fixed with 1 mL of paraformaldehyde per well for 20 min in the dark. After cell fixation, paraformaldehyde was removed and the fixed cells were washed three times with PBS. Following this protocol, the cell nuclei were stained with DAPI dye at a concentration of 1 μL dye per mL of PBS for 30 min in the dark. The cells were then washed three times with PBS to remove any remaining DAPI dye, and 1 mL of PBS was left in each well. The fixed cell samples were examined under an inverted life science microscope (Olympus-Xcellence RT Life Science Microscopy). Two different filters were used for microscopic imaging: one specific for the DAPI dye region (λ_exc_: 352–402 nm and emission: 417–477 nm) and another for the NIR region (λ_exc_: 550 nm and emission: 650 nm long pass) to capture images of the cellular nucleus and NIR-emitting QDs. As a control, the same experimental procedure was performed on control cells with no QDs.

### 2.7. Preparation of plasmid DNA and polyplex formation

The pBI-MCS-EGFP plasmid DNAs were obtained using an endotoxin-free Qiagen HiSpeed Plasmid Maxi Kit (25) (Valencia, CA, USA) after being propagated in *Escherichia coli* DH5a cells. A UV–vis spectrophotometer (Nanodrop-1000, ND1000) was used to measure the concentration and purity of the purified pDNA after it had been dissolved in Tris-EDTA (TE) buffer solution. Plasmid DNA (pDNA) and Ag_2_S@2MPA/LPEI QDs polyplex were prepared with various N/P ratios. For this purpose, the nitrogen percentage of Ag_2_S@2MPA/LPEI QDs was calculated using a thermogravimetric analyzer (TGA) and organic CHNS/O elemental analysis. Ag_2_S@2MPA/LPEI QDs were dissolved and filtered in 20 mM Hepes Buffer Glucose (HBG; 5% and pH 7.4) to a final amount of 1 mg/mL of total QDs. Polyplexes were formulated at different N/P (0.7:1, 1.5:1, 2.5:1) ratios by dissolving known amounts of QDs in 25 μL of HBG and binding 1 μg of pDNA again in 25 μL of HBG by first pipetting several times and then incubating for 15 min at room temperature. Agarose gel electrophoresis was applied to determine the ability of Ag_2_S@2MPA/LPEI QDs to condense pDNA and retard the migration of nucleic acids. Measurements were normalized to that of free pDNA and 25 kDa LPEI was used as the control delivery system.

### 2.8. Gene transfection

Cell transfection was performed on different cell lines. HEK 293T cells, HeLa cells, and MEF p53−/− cells were cultured 5 × 10^4^ cell/well in complete DMEM and HCT 116 wt and HCT 116 p53−/− cells were cultured in McCoy’s 5A Medium at a 20 × 10^4^ cells/well in 6-well plates. The cells were grown to 60% confluence at the time of treatment overnight in a humidified 37 °C environment in 5% CO_2_. Ag_2_S@2MPA/LPEI QDs were first diluted to a concentration of 1 mg/mL in HEPES buffered glucose (5%) (HBG). N/P optimization was performed at different (10:1, 20:1, 30:1, and 40:1) ratios with a final volume of 2 mL formulated by mixing equal volumes of QD and GFP-pDNA containing HBG solution and then further diluting with fresh medium. After removing the previous growth medium from the cells, the complexes were added. For the purpose of cell imaging investigations, the medium was changed after 4 h and replaced with new medium containing 10% FBS. The incubation period was then extended to 24 h. Following this 24-h period, the growth medium was taken out and the cells were examined. To analyze the transfection efficiency of the QDs, specific filters in the GFP region (λexc: 385–415 nm and emission: 515–535 nm long pass) were used. The same experimental procedure was carried out with control cells that were either treated with Ag_2_S@2MPA/LPEI QDs or solely free LPEI as a means of controlling plasmid transfection.

## 3. Results and discussion

### 3.1. Synthesis of 2MPA/LPEI-coated Ag_2_S (Ag_2_S@2MPA/LPEI) NIR QDs

According to previous research, linear PEI is a more effective transfecting polymer than branched ones and is less harmful (Wiseman et al., 2003). In the present study, biocompatible, cationic, NIR emitting theranostic QDs were reproduced and their optical imaging efficiencies and gene transfection were evaluated. The ratio of coating materials/Ag/S mole was established at 20/4/1. The mole ratio of N of PEI to the SH groups of 2MPA remained at 20/80.

### 3.2. In vitro cell viability/cytotoxicity studies

**Figure 2:**
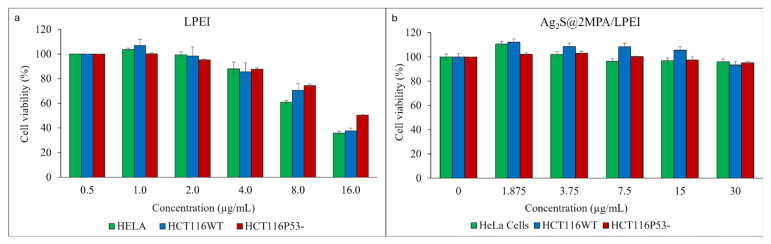
Cell viability of HeLa cancer cell lines treated with a) LPEI and b) Ag_2_S@2MPA/LPEI.

Ag_2_S@2MPA/LPEI was investigated for cytotoxicity in vitro. In contrast, free LPEI was also subjected to a viability test. The viability test on HeLa cells demonstrated that Ag_2_S@2MPA/LPEI did not induce a significant cytotoxic effect up to 30 μg/mL. The free LPEI was safe only when the concentration decreased to 4 μg/mL and the toxicity was calculated as 60% and 30% for doses of 16 μg/mL and 8 μg/mL free LPEI, respectively (Figure 2). Although free LPEI showed two times less toxicity compared to free BPEI, it seems extremely toxic for biological application (Duman et al., 2015). Binding LPEI to the surface of Ag_2_S QDs improved cytocompatibility dramatically with respect to free LPEI. This significant difference in cytocompatibility between Ag_2_S@2MPA/LPEI and free LPEI may be partially due to the reduction of free amine groups following the amine groups’ adsorption onto the QDs’ crystal surface. Indeed, there were at least two improvements in the cytotoxicity of Ag_2_S@2MPA/LPEI compared with Ag_2_S@2MPA/BPEI QDs that were previously developed by Duman et al. (2015). This result is compatible with the results for free LPEI and BPEI.

### 3.3. In vitro cell internalization and optical imaging of Ag_2_S@2MPA/LPEI

**Figure 3:**
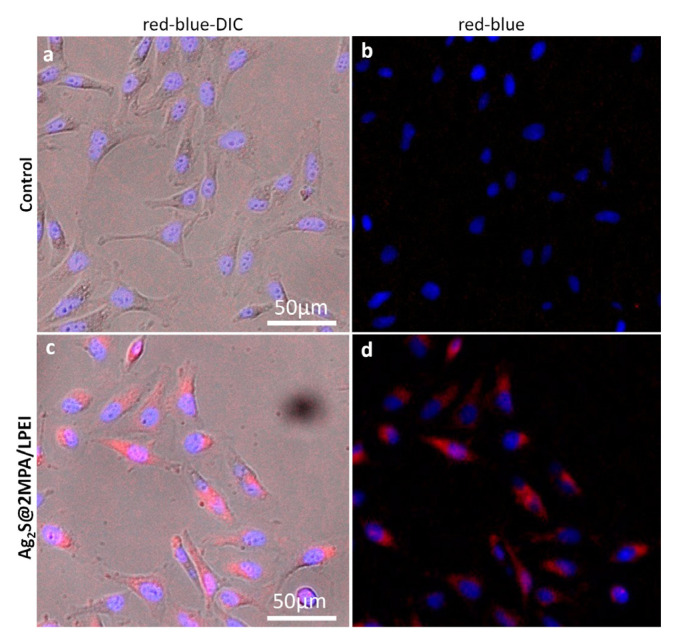
Fluorescent microscopy images of control HeLa cells (a & b) and treated HeLa cells incubated with free cell medium (a & b) and 50 μg/mL Ag_2_S@2MPA/LPEI QDs containing medium (c & d) for 4 h. NIR filter (excitation wavelength 550 nm, emission 640 nm long pass filter).

A delivery method must meet several essential requirements in order to be considered an outstanding theranostic instrument, including strong NIR emission, low cytotoxicity, and effective gene transport capability. Robust intracellular signals derived from QDs are crucial for diagnostic applications. For optical imaging, strong intracellular signals coming from QDs are crucial (Luker and Luker, 2008). HeLa cells treated with QDs were observed under an inverted fluorescent microscope to visually validate the internalization of Ag_2_S@2MPA/LPEI QDs by the cells and assess their use as optical diagnostic probes for diagnosis (Figures 3a–d).

Although the sensitivity of the detectors utilized in the microscope goes down around 30% immediately after 800 nm, owing to the application of a specific NIR filter, Ag_2_S@2MPA/LPEI QD demonstrated a very strong signal at a maximum emission peak of 897 nm. Ag_2_S@2MPA/LPEI QDs were found to have a very strong optical signal when the images of internalized QD cells were compared to those of control cells. The QD signal was dispersed throughout the cytoplasm of the cells. In Figures 3c and d, the blue color depicts the cell nucleus, which is photographed by the DAPI filter, and the red color indicates the emission coming from Ag_2_S@2MPA/LPEI QDs using the NIR filter. Ag_2_S@2MPA/LPEI QDs exhibit intense luminosity and effective cell internalization with endosomal localization as shown in Figure 3, indicating their significant potential as an optical probe.

### 3.4. Plasmid loading on Ag_2_S@2MPA/LPEI QDs

The basic components of the complex, plasmid DNA and Ag_2_S@2MPA/LPEI, are required to be clearly defined in order to analyze DNA/PEI complexation. According to the thermogravimetric analysis results, Ag_2_S@2MPA/LPEI QDs has 49.9% organic content, which was compatible with the CHNS/O elemental analysis (N: 9.6%, C: 25.9%, H: 4.9%, and S: 9.5%) results. According to the CHNS/O elemental analysis results the weight percentage of nitrogen atoms in the organic contents of Ag_2_S@2MPA/LPEI QDs was about 9.6%. Moreover, 1 μg of DNA contains 3 nmol of phosphate (Boussif et al., 1995). Therefore, to form polyplexes at N/P 1 with 1 μg of DNA, 0.46 μg of Ag_2_S@2MPA/LPEI QDs was required.

An earlier description of the N/P ratio computation for complex formation using linear PEI was given (Boussif et al., 1995; Moreno et al., 2022). The mass per charge ratio of DNA and PEI serves as the basis for the computation. DNA contains one anionic charge (P) for every 330 g mol^−1^, whereas PEI has one cationic charge for every 44 g mol^−1^. Thus, 0.13 μg of LPEI was needed to generate polyplexes at N/P 1 with 1 μg of DNA (Perevyazko et al., 2012; Moreno et al., 2022).

**Figure 4:**
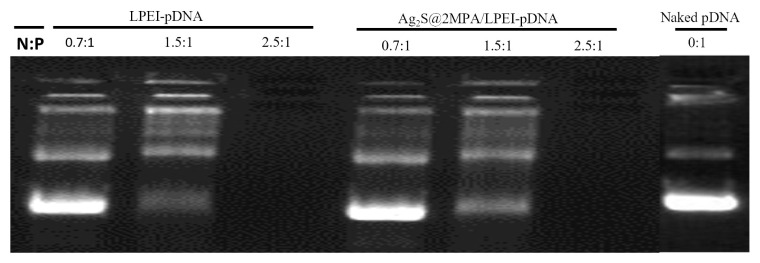
Agarose gel electrophoresis retardation assay of 25 kDa LPEI and Ag_2_S@2MPA/LPEI QDs at different N/P ratios. Naked plasmid DNA and free LPEI polymer were used as controls.

**Figure 5:**
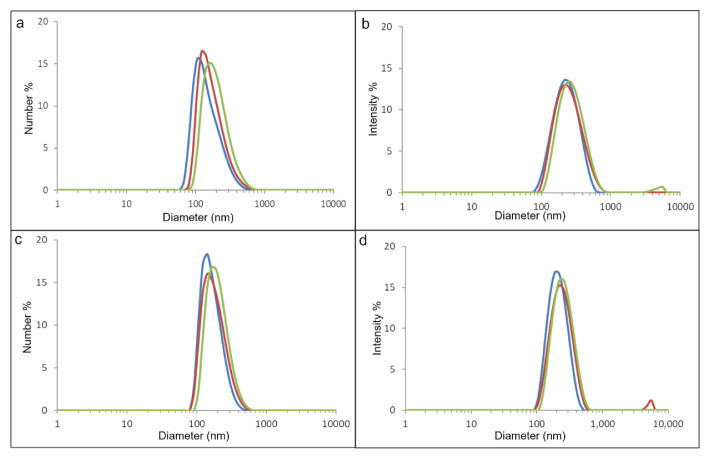
The logarithmic size (by number and intensity) of plasmid loaded Ag_2_S@2MPA/LPEI at N/P ratios of 10 (a and b) and 20 (c and d). Size peaks in different colors (red, blue, and green) represent triplicate measurement of the samples.

According to the agarose gel electrophoresis results, linear PEI is slightly more effective in condensing and retarding pDNA. Although at an N/P ratio of 1.5 linear PEI could condense most of the pDNA, Ag_2_S@2MPA/LPEI QDs could just condense around 50% of it (Figure 4). Free DNA was not seen to have migrated into the gel at an N/P ratio of ≥2.5, indicating that Ag_2_S@2MPA/LPEI QDs and linear PEI had fully complexed the plasmid DNA. Although application of the N/P ratio at 2.5 for both linear PEI and Ag_2_S@2MPA/LPEI QDs resulted in full condensation and total retardation of the migration of pDNA, for the formation of stable polyplex with acceptable zeta potential we needed to increase the N/P ratio to at least 5 (D’Andrea et al., 2014). Therefore, for gene transfection studies an N/P ratio of 5 was applied as the minimum ratio. Dynamic light scattering (DLS) analysis of Ag_2_S@2MPA/LPEI-pDNA at N/P ratios of 10 and 20 showed similar results and was calculated as 140 ± 20 nm by number and 220 ± 30 nm by intensity. However, the plasmid loading at an N/P ratio of 20 resulted in smaller and narrower particle size distribution than at a ratio of 10 (Figure 5).

### 3.5. Ag_2_S@2MPA/LPEI QDs as in vitro transfection agents

**Figure 6:**
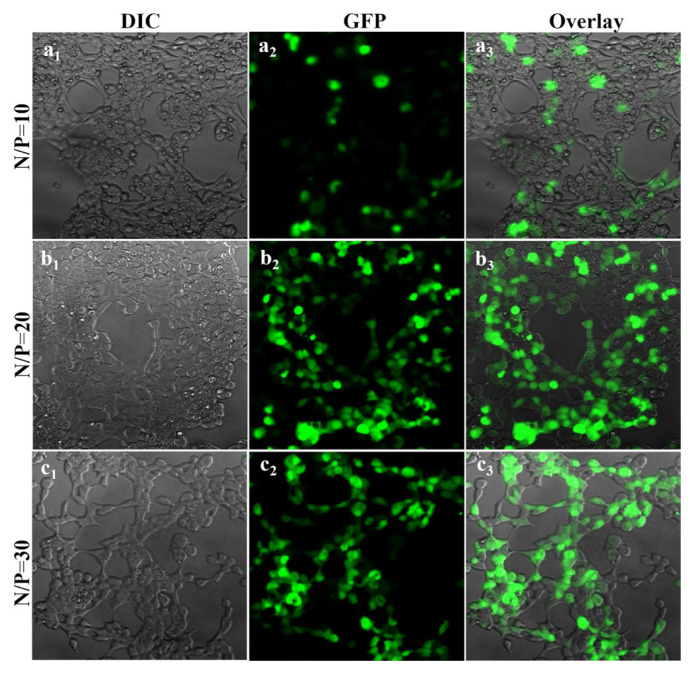
Fluorescence images (200×) of HEK 293T cells after incubation with medium containing Ag_2_S@2MPA/LPEI loaded with 1 μg/mL GFP plasmid DNA concentration at N/P ratios of 10 (a_1_, a_2_, and a_3_), 20 (b_1_, b_2_, and b_3_), and 30 (c_1_, c_2_, and c_3_).

**Figure 7:**
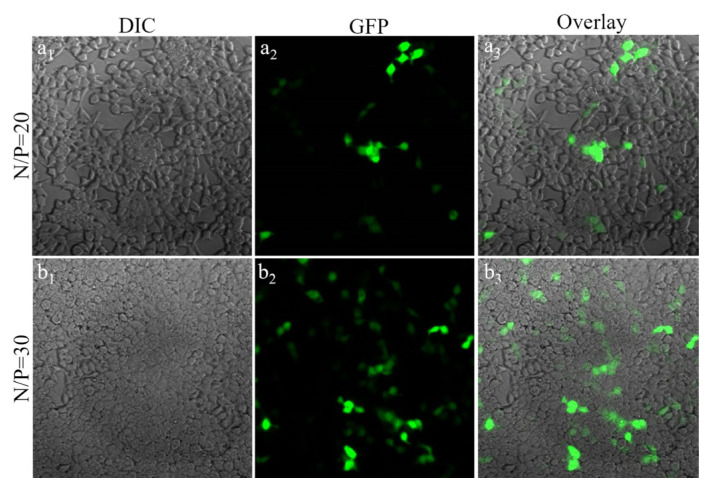
Fluorescence images (200×) of HEK 293T cells after incubation with medium containing 25 kDa LPEI loaded with 1 μg/mL concentration of GFP plasmid at N/P ratios of 20 (a_1_, a_2_, and a_3_) and 30 (b_1_, b_2_, and b_3_).

The green fluorescent protein (GFP) gene, a common standard proof-of-concept material used in cancer gene therapy, was used in transfection efficiency research to evaluate the transfection capacity and efficiency of the manufactured theranostic system. First, the transfection efficiency of GFP plasmid with Ag_2_S@2MPA/LPEI was optimized on highly transfectable HEK 293T cells at N/P 10, 20, and 30 ratios (Figures 6 and 7) and the outcomes were contrasted using a standard transfection agent (25 kDa LPEI) according to the normal methodology.

HEK 293T cells, which were produced by incorporation of a human adenovirus 5 (AD5) fragment into chromosome 19 of the host human embryonic kidney genome, are suitable transfectable cells and have been successfully utilized to overexpress a wide variety of not only model proteins but also native human proteins (Graham et al., 1977; Thomas and Smart, 2005). The transfection effectiveness with Ag_2_S@2MPA/LPEI QDs was extremely poor at an N/P ratio of 10, but it increased to a maximum level when the N/P ratio was raised to 20. This was shown by inverted microscope images of transfected cells. The results demonstrated that further increasing the N/P ratio in Ag_2_S@2MPA/LPEI QDs did not increase the transfection efficiency any further. Therefore, an N/P ratio of 20 was chosen as optimal to be applied for gene transfection with Ag_2_S@2MPA/LPEI (Figure 6).

Considering transfection with free linear PEI, transfection was very low at an N/P ratio of 20. The linear PEI transfection efficiency increased at a ratio of 30 N/P. Ag_2_S@2MPA/LPEI QDs had a significantly greater capacity for transfection, even at an N/P ratio of 20 (Figures 6 and 7). The density and luminance of expressions of GFP belonging to Ag_2_S@2MPA/LPEI-pGFP were higher than those of LPEI-pGFP. Therefore, according to the results of transfection experiments on HEK 293T cells, it can be inferred that application of Ag_2_S@2MPA/LPEI QDs as a delivery system not only decreased the cytotoxicity of the delivery system but also resulted in a much higher transfection level.

**Figure 8:**
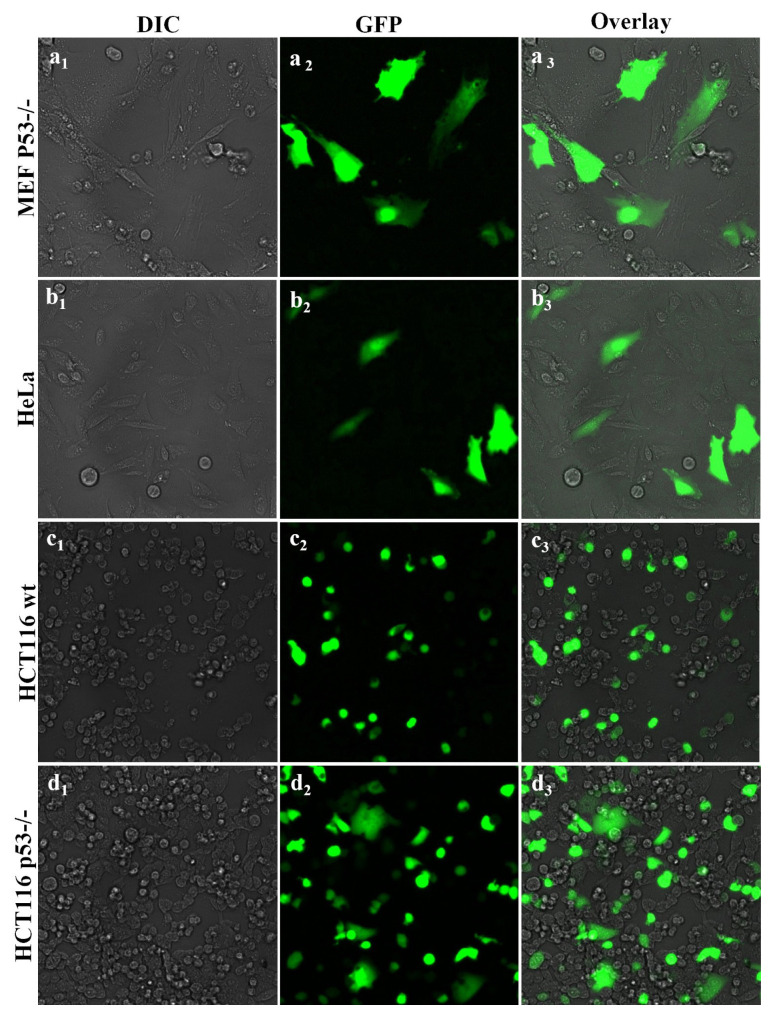
Fluorescence images (200×) of MEF p53−/− (a_1_, a_2_, and a_3_), HeLa (b_1_, b_2_, and c_3_), HCT116 wt (c_1_, c_2_, and c_3_), and HCT116 (p53−/−) (d_1_, d_2_, and d_3_) cells after incubation with medium containing with Ag_2_S@2MPA/LPEI loaded with 1 μg/mL GFP plasmid DNA concentration at N/P ratios of 20.

Following optimization of the N/P ratio as 20 on highly transfectable HEK 293T cells, the GFP plasmid transfection ability of Ag_2_S@2MPA/LPEI with GFP plasmid was tested on different cancer cell lines, namely MEF p53−/−, HeLa, HCT116 wt, and HCT116 p53−/− cancer cells (Figure 8). The density and luminance of green fluorescence belonging to pGFP on different cancer cells were less than those of HEK 293T cells, which are genomically modified highly transfectable model cells. According to these fluorescence image results Ag_2_S@2MPA/LPEI theranostic QDs turned out to be highly suitable to be applied for the transfection of both HCT116 wt and HCT116 p53−/− cells. It seems that transfection was slightly more effective on p53 knockout cells than on wild-type HCT116 cells. From the applied cell lines, the MEF cells were the least effectively transfected cells, which is expected due to the fact that they are challenging primary cells to transfect (Lee et al., 2017). These results confirmed the N/P ratio of 20 as the most effective value for the application of Ag_2_S@2MPA/LPEI QDs as gene delivery vectors for cancer cells.

## 4. Conclusion

Theranostics systems simultaneously applicable as gene/drug delivery and imaging agents have become very popular in the last decade. One of the most useful medical innovations could be the combination of near-infrared optical imaging and near-infrared emitting quantum dots for gene transfer. Ag_2_S@2MPA/LPEI, newly created cationic silver sulfide QDs with a strong NIR luminescence, were shown in our study to be promising theranostic systems for gene transport and diagnostic uses. Application of Ag_2_S semiconductor QDs instead of highly toxic heavy metal QDs as a core has great importance in the development of cytocompatible QDs. An NIR fluorescence microscope was used to see significant intracellular NIR luminescence, which validated the quantum dots’ optical imaging and diagnostic capabilities. Application of LPEI instead of BPEI (in combination with 2MPA) as a coating for QDs has a significant contribution in terms of cytocompatibility. The cytocompatibility of delivery systems can also be achieved through PEGylation. However, due to interference with gene delivery potential and endosomal escape PEGylation of gene delivery system generally is not preferred. Here, the GFP standard gene was used to transport a gene using the cationic nature of Ag_2_S@2MPA/LPEI QDs. Transfection efficiency was greater when using Ag_2_S@2MPA/LPEI QDs in comparison to conventional LPEI transfection reagents. The cytocompatible Ag_2_S@2MPA/LPEI QDs that are generated are highly successful in delivering genes and have a strong NIR emission, which makes them useful for theranostic applications. These findings suggest that Ag_2_S@2MPA/LPEI can be an effective theranostic gene carrier system to deliver genes and being tracked for improved cancer therapy and imaging studies.
